# The Association between Residence in a Food Desert Census Tract and Adherence to Dietary Patterns in the REGARDS Cohort

**Published:** 2018

**Authors:** Marquita S. Gray, Sindhu Lakkur, Virginia J. Howard, Keith Pearson, James M. Shikany, Monika Safford, Orlando M. Gutiérrez, Natalie Colabianchi, Suzanne E. Judd

**Affiliations:** 1 Department of Biostatistics, University of Alabama at Birmingham (UAB), Birmingham, AL, USA; 2 Department of Epidemiology, UAB, Birmingham, AL, USA; 3 Nutrition and Dietetics, Samford University, Birmingham, AL, USA; 4 Department of Medicine, UAB, Birmingham, AL, USA; 5 Division of General Internal Medicine, Weill Cornell Medical College, New York, NY, USA; 6 School of Kinesiology, University of Michigan, Ann Arbor, MI, USA

**Keywords:** Food desert, Census tract, Dietary patterns, Diet

## Abstract

Increased interest in determining areas in need of improved food access led the U.S Department of Agriculture (USDA) to define food desert census tracts; however, no nationwide studies have compared dietary patterns in food desert tracts to other tracts. Our objective was to examine dietary patterns in residents of food desert and non-food desert census tracts. We performed a cross-sectional analysis of 19,179 participants in the REasons for Geographic and Racial Differences in Stroke (REGARDS) study enrolled January 2003-October 2007. We used participants’ geocoded address with USDA Food Desert Locator to identify food deserts and multivariable-adjusted odds ratios (ORs) to calculate adherence to Southern, Plant-based, and Mediterranean dietary patterns. Odds of adherence to the Southern dietary pattern were higher among white high school graduates (OR=1.41; 95% CI: 1.20–1.67), white college graduates (OR=1.91; 95% CI: 1.55–2.35) and black college graduates (OR=1.38; 95% CI: 1.14–1.68) who reside in a food desert versus non-food desert. Odds of adherence to the Plant-based dietary pattern were 15% lower among non-southeastern residents (OR=0.85; 95% CI: 0.72–0.99), who reside in food desert versus non-food desert. No statistically significant differences were observed for the Mediterranean dietary pattern. Residents living in food deserts had lower adherence to healthy dietary pattern than residents not living in food deserts; the association may vary by race, education, and region.

## 1. Introduction

Approximately 23.5 million Americans live in low-income neighborhoods located more than one mile from a supermarket [1]. Increased interest in determining areas in need of improved food access led the US Department of Agriculture (USDA) to define food desert census tracts to identify areas that would benefit from increased access to healthy foods with high nutrient density [1]. The USDA’s Economic Research Service defines food deserts as census tracts that have both low income and low access to supermarkets. Using this definition, the USDA classified 6,529 of all 64,999 census tracts in the continental US as food deserts based on 2000 Census data and 2006 data on supermarket locations [2].

The role of the food environment in contributing to health disparities is supported by many population-based studies and is the subject of several reviews [3–5]. Using a variety of measures to define the food environment, many of these studies found that access to supermarkets is associated with healthier dietary intake [5–7]. According to the Atherosclerosis Risk in Communities (ARIC) study, for each additional supermarket within a census tract, the number of black and white adults meeting national guidelines for fruit and vegetable consumption was 32% and 11% higher, respectively [7]. Other studies have examined the food environment and comprehensive dietary quality using a priori defined dietary scores. In the Multi-Ethnic Study of Atherosclerosis (MESA), compared to those who lived in areas with the highest supermarket density, those who lived in areas with the lowest density of supermarkets were 25% less likely to have a healthy diet (defined as the top quintile of the Alternate Healthy Eating Index) [6].

Nutrition researchers have been well accepting of the idea that dietary patterns may serve as better predictors of health than intakes of individual nutrients or foods [8]. In the nationwide REasons for Geographic and Racial Differences in Stroke (REGARDS) cohort, the Mediterranean dietary pattern and two empirically-derived patterns (Plant-based and Southern) have been associated with cognitive impairment/function [9, 10], stroke risk [11], and incident coronary heart disease [12]. Additionally, empirically derived dietary patterns are unique because they may also capture a complex set of socio-cultural determinants of diet [11]. More importantly and perhaps more interestingly, understanding the association between the food environment and adherence to empirically derived dietary patterns can identify populations where additional supermarkets and behavioral interventions may be necessary.

Given that previous studies of the food environment used different definitions of food access and were restricted to smaller geographic regions, it is unknown how diets in USDA defined food desert census tracts compare to those in other tracts across the US. Furthermore, it is unknown whether the role of availability of healthy foods in dietary pattern adherence varies by demographic characteristics in a nationwide heterogeneous population. In the current study, we examined the association between living in a food desert census tract and a priori and a posteriori-defined dietary patterns using data from the REGARDS study.

## 2. Methods

### 2.1. Study Participants

REGARDS is a prospective cohort study designed to examine racial and regional differences in stroke across the continental United States. Details of the study design can be found elsewhere [13]. Briefly, a total of 30,239 black or white participants, ages 45 or older, were enrolled from January 2003 to October 2007. Commercially available lists from Genesys, Inc. (Daly City, CA) were used for recruitment. Participants from 1,842 (59%) of 3,140 US counties were randomly contacted by telephone and mail, with an oversampling of blacks and residents of the Stroke belt (Alabama, Arkansas, Georgia, Louisiana, Mississippi, North Carolina, South Carolina, and Tennessee) and buckle (the coastal plain of North Carolina, South Carolina, and Georgia). The telephone response was 33% and 49% agreed to cooperate. After verbal consent, a baseline telephone interview was conducted to collect demographic data and medical history. Written informed consent, physical measurements, and biological samples, were obtained during an in-home visit. Self-administered questionnaires were left with the participant to be returned by self-addressed, prepaid envelopes. The institutional review boards of the multiple participating entities approved this study.

### 2.2. Dietary Assessment

Diet was assessed by a self-administered Block 98 Food Frequency Questionnaire (FFQ) [14], which participants were instructed to complete at the end of their home visit. Participants reported diet in the past year and returned the completed FFQ within 3 months of the home visit. Mean daily intake (grams) of individual foods on the FFQ were estimated by NutrtionQuest (Berkeley, CA). The Block 98 FFQ has been validated in other studies with similar populations [15, 16].

### 2.3. Dietary Pattern Identification

In a previous study, dietary patterns were derived empirically in this population using principal components analysis [11]. Five dietary patterns were identified: Southern, Plant-based, Convenience, Sweets/Fats, and Alcohol/Salads. Additional analyses revealed that the Southern and Plant-based dietary patterns were associated with health outcomes; therefore, these two patterns were studied here [11, 12]. Participants received scores for their adherence to each dietary pattern, where higher scores indicated stronger adherence. The Southern dietary pattern was characterized by high factor loadings of fried foods, added fats, eggs and egg dishes, sugar-sweetened beverages, organ meats, and processed meats [11]. The Plant-based pattern consisted of high factor loadings of vegetables, fruits, fish, and beans [11].

We also calculated the Mediterranean Diet (MeD) score, which was calculated by assigning 0 or 1 point to each of nine a priori defined dietary components, based on intakes above and below the median intake [9]. The components include fruits, vegetables, legumes, cereals, fish, meat, dairy products, the ratio of monounsaturated fats to saturated fats, and alcohol consumption. The MeD score assignment scheme is shown in Table 1. The points assigned to each component were summed to create the overall MeD score. A higher score indicates stronger adherence to a Mediterranean-type diet.

### 2.4. Food Desert Census Tract Residence

Using SAS statistical software version 9.4 (SAS Institute, Cary, NC), participants were geocoded based on baseline address. Residence in a food desert census tract was obtained from the USDA Food Desert Locator [17]. Tracts that met the U.S. Treasury Department’s New Markets Tax Credit (NMTC) program eligibility criteria were classified as low income. To qualify as a tract with low access to healthy food, at least 33% of the population, or at least 500 people, had to have low access to healthy food—living more than 1 mile away from a grocery store or supermarket in urban tracts, and 10 miles away in rural tracts [17]. Two national directories were used to identify supermarkets and grocery stores, which were defined as stores with revenue of over $2 million and containing all the major food departments [1].

### 2.5. Statistical Analysis

For descriptive statistics, the means, standard deviations, and frequencies were calculated for covariates by food desert census tract residence. To examine differences in demographic characteristics by food desert census tract residence, the chi-square test was used for categorical variables and the t-test was used for continuous variables.

High adherence was defined as scoring in the top quartile of the respective dietary pattern/score (Southern, Plant-or Mediterranean) in the overall REGARDS analytical based, population. Multivariable-adjusted odds ratios (ORs) and their corresponding 95% confidence intervals (CIs) were calculated using logistic regression models. Covariates included age, sex, race (black, white), household income (less than $20k, $20–34k, $35–74k, 75k and above), highest level of educational attainment (less than high school, high school graduate, college graduate or above), region (southeastern, non-southeastern), Rural-Urban Commuting Area (RUCA) code (isolated, small rural, large rural, urban), and relationship status (married, divorced, other, single, widowed). Models were examined for collinearity among independent variables. Interactions between food desert residence and covariates (age, race, sex, income, education, and region) were assessed using the likelihood ratio test. Evidence of interaction was determined by an a priori α level of 0.10. When we observed significant interaction, we conducted stratified analyses to determine whether the food desert-dietary pattern association was modified by the covariate. All analyses were conducted using SAS version 9.4.

## 3. Results

Of the 30,239 REGARDS participants, we excluded participants who: (1) had missing baseline forms (n=56), (2) could not be geocoded to a census tract (n=2,906), (3) did not have complete information to derive dietary patterns (n=8,090), or (4) had missing covariate information (n=8). This resulted in an analytical cohort of 19,179 participants. Excluded subjects were more likely to be low-income, black, unmarried males with lower educational attainment.

Table 2 presents the demographic characteristics of participants in the analysis cohort for residence versus non-residence in a food desert census tract. Compared to participants who did not reside in a food desert, participants who resided in a food desert were more likely to be black, unmarried women living in the Southeastern United States with an annual household income of less than $34,000 and less than a high school education. No significant difference in age by food desert residence was observed.

Associations between residence in a food desert and adherence to each of the dietary patterns are shown in Table 3. For the Southern dietary pattern, there were statistically significant interactions between food desert residence and race (p=0.0005) and food desert residence and educational attainment (p=0.003), so we presented these analyses separately by educational attainment within each race (Table 3).

The odds of adherence to a Southern diet pattern were 38–91% higher among white high school graduates (OR=1.41; 95% CI: 1.20–1.67), white college graduates (OR=1.91; 95% CI: 1.55–2.35) and black college graduates (OR=1.38; 95% CI: 1.14–1.68) who lived in a food desert compared to those who did not. None of the ORs for black high school graduates and blacks and whites with less than a high school education were statistically significant.

In the Plant-based dietary pattern, a statistically significant interaction was observed between food desert and region (p=0.03); these analyses are presented separately for the southeastern and non-southeastern region of the United States. The odds of adherence to a Plant-based dietary pattern were 15% lower among food desert residents com-pared with residents of non-food deserts outside the south-east (OR=0.85; 95% CI: 0.72–0.99), but not significantly different among southeastern residents (OR=1.05; 95% CI: 0.93–1.18).

There were no significant interactions for the MeD, however, we present stratified results to be congruent with presentation of other dietary patterns (Table 3). Among all participants, the odds of adherence to a MeD pattern were 14% lower (OR=0.86; 95% CI: 0.78–0.94) in those who resided in a food desert compared to those who resided in other census tracts.

## 4. Discussion

We examined the association between residence in a food desert and adherence to dietary patterns in a nationwide cohort, hypothesizing that residence in a food desert would be associated with increased adherence to a Southern dietary pattern and decreased adherence to a Plant-based and MeD pattern. While our findings support this hypothesis, these associations also varied by race and educational attainment for the Southern dietary pattern. The association between food desert residence and adherence to the Plant-based dietary pattern varied by region. An examination of the a priori defined MeD pattern found that living in a food desert was inversely associated with adherence to the MeD pattern for all participants, without observed differences in race, education or region. A larger proportion of those living in food deserts adhered to the Southern dietary pattern compared to those living in other census tracts. However, the proportion of participants adhering to the Plant-based or MeD patterns were similar when examined by food desert residence.

Adherence to the Southern, Plant-based, and MeD dietary patterns have previously been shown to be associated with different health outcomes in the REGARDS cohort [9, 11, 12]. Participants in the highest quartile of adherence to the Southern dietary pattern had a 39% increased risk of stroke, relative to the lowest quartile of adherence [11]. The same study found that compared to participants in the lowest quartile of adherence to the Plant-based pattern, participants in the highest quartile of adherence had a 29% decreased risk of stroke [11]. Among non-diabetic participants, high adherence to the MeD pattern was associated with 27% higher odds of cognitive impairment relative to low adherence [9]. A meta-analysis found that a 2-point increase in MeD score was associated with an 8% lower risk of all-cause mortality and a 10% lower risk of CVD [18]. A large, randomized controlled trial recently reported that the Mediterranean dietary pattern reduced the risk of CVD by 29% [19].

We observed that the association between residence in a food desert and adherence to the Southern dietary pattern differed by race, which may be explained by cultural preferences. In the REGARDS population, blacks were 5 times more likely to adhere to a Southern dietary pattern compared to whites (data not shown) [11]. The southern dietary pattern may be an integral part of cultural identity for blacks, resulting in high prevalence of this type of diet regardless of where blacks live. A similar effect modification by race was observed in the National Health and Nutrition Examination Survey III (NHANES III) study of neighborhood socioeconomic status and fruit and vegetable consumption; the magnitude of association was higher among whites compared to blacks [20]. In Pollard’s theoretical framework of food choice, availability of foods and familiarity with foods (acquired though long-term habits and cultural traditions) are two of the major determinants of food choice [21]. Among blacks, the influence of cultural factors on food choice may be stronger than the influence of healthy food availability, suggesting that merely making healthier foods available in food deserts may not be sufficient to encourage healthier diets among blacks [22, 23].

In addition to race, we also found that educational attainment modified the association between residence in a food desert and adherence to the Southern dietary pattern, an observation supported by other studies. [20–23]. Educational attainment influences many determinants of dietary intake, such as dietary knowledge, cultural dietary norms, food preparation skills, and employment [24]. A review of cross-sectional studies concluded that higher quality diets tend to be consumed by those with higher levels of education [25]. Our study found that for blacks and whites with low levels of educational attainment, adherence to the Southern dietary pattern was not appreciably different between residents in food deserts and non-food deserts. Longitudinal studies in populations with low proportions of college graduates have also reported little association between proximity to a supermarket and fruit and vegetable consumption; this was observed in the Coronary Artery Risk Development in Young Adults (CARDIA) study [26] and another longitudinal study that examined the impact of opening a new supermarket on fruit and vegetable intake in a Philadelphia food desert [27]. However blacks and whites with high levels of educational attainment who lived in food deserts were more likely to adhere to a Southern dietary pattern compared to those with high levels of educational attainment who did not lived in food deserts.

One of the strengths of our study was the examination of empirically identified dietary patterns, in addition to an a priori defined dietary pattern. We observed a significant region-food desert interaction in the association between food desert residence and adherence to the Plant-based pattern, but not the MeD pattern, suggesting that there may be nuanced differences in the “healthy” dietary pattern that are captured by factor analysis. Another strength of our study was that our participants were sampled from a large, nationwide population-based cohort. There was considerable variability of sociodemographic characteristics among the participants. Other cross-sectional studies in the US have examined the role of food access and diet, finding that among all participants low food access is associated with a poor diet. These studies were smaller and had populations with less geographic and socioeconomic diversity [6, 7].

Several limitations of this study should be acknowledged. For a census tract to be defined as a food desert by the USDA, it must meet the criteria of being low income and have low access to healthy food. Since the definition of a food desert census tract also considers income, in addition to food access, our findings may not be comparable to other studies that use different criteria to define a food desert. The generalizability of our findings to other populations may be limited because our cohort includes only black and white participants. To compare our population to others, we also examined the adherence to a Mediterranean dietary pattern. Participants who scored in the top quartile of MeD score were defined as having high adherence to the MeD pattern. Although we used a quartile approach for high adherence to MeD, we understand that this may attenuate these associations. However, our highly adherent participants had a MeD score ranging from 6–9 points, which is also consistent with other studies that have defined high adherence to MeD as a score of 6–9 points [9, 28, 29]. Unfortunately, other studies do not have clear cut points for what is considered high adherence for a Southern and a Plant-based dietary pattern. Therefore, the quartile approach would be more appropriate for these dietary patterns. Our study also had a number of participants with missing geocode and dietary information, who were not included in this analysis. These excluded participants who were more likely to be unmarried black males with lower educational attainment and income; however, our analysis still included a substantial number of participants in the aforementioned subgroups.

In summary, our findings demonstrate that the association between residence in a food desert and adherence to dietary patterns may vary by race, education, and region. Perhaps the most interesting finding in this study is that residence in a food desert census tract has strong associations with adherence to dietary patterns; regardless of educational attainment or being in the top quartile for adherence, residents in a food desert census tract can still have a poor diet. This is particularly relevant to policymakers. Changing dietary patterns in the US requires a multifaceted approach and improving food access is just one step in the process. Culturally sensitive and community-driven interventions to changing dietary behavior may be the best approach to making diets healthier in the US, but making healthy food available (respective of where one lives) is a needed first step.

## Figures and Tables

**Table 1.** Mediterranean Diet Score Assignment Scheme

Mediterranean Diet Component	Score Assignment
Fruits	0=Below median, 1= At or above median
Vegetables	0=Below median, 1= At or above median
Legumes	0=Below median, 1= At or above median
Cereals	0=Below median, 1= At or above median
Fish	0=Below median, 1= At or above median
Meat	1=Below median, 0= At or above median
Dairy Products	1=Below median, 0= At or above median
Ratio of monounsaturated fats to saturated fats	1=Belowmedian, 0=Atorabove sex-specific median
Alcohol^a^	1=Moderate consumption, 0=Zero or above moderate consumption

a Women:0–7 drinks/week, Men: 0–14 drinks/week

**Table 2.** Descriptive characteristics by residence in a food desert census tract in the REGARDS Study, 2003–2007^a^

Participant characteristics	Not a food desert (n=16,294)	Food desert (n=2,885)	p-value
Age, y, mean (SD)	64.9 (9.3)	64.7 (9.1)	0.29
Sex, n (%)			<0.01
Male	7,287 (44.7)	1,170 (40.6)	
Female	9,007 (55.3)	1,715 (59.5)	
Race, n (%)			<0.01
Black	4,921 (30.2)	1,644 (57.0)	
White	11,373 (69.8)	1,241 (43.0)	
Income, n (%)			<0.01
Less than $20k	2,306 (14.2)	707 (24.5)	
$20k–34k	3,800 (23.3)	781 (27.1)	
$35k–$74k	5,224 (32.1)	800 (27.7)	
$75k and above	3,030 (18.6)	273 (9.5)	
Refused	1,934 (11.9)	324 (11.2)	
Education, n (%)			<0.01
Less than high school	1,389 (8.5)	437 (15.2)	
High School graduate	8,453 (51.9)	1,627 (56.4)	
College graduate or above	6,452 (39.6)	821 (28.5)	
Region, n (%)			<0.01
Southeast^b^	8,746 (53.7)	1,887 (65.4)	
Outside the southeast	7,548 (46.3)	998 (34.6)	
RUCA, n (%)			0.03
Isolated or Small rural	1,453 (8.9)	258 (8.9)	
Large rural	2,033 (12.5)	310 (10.8)	
Urban	12,808 (78.6)	2,317 (80.3)	
Relationship Status, n (%)			<0.01
Married	10,236 (62.8)	1,534 (53.2)	
Divorced	2,232 (13.7)	480 (16.6)	
Other	276 (1.7)	80 (2.8)	
Single	817 (5.0)	181 (6.3)	
Widowed	2,733 (16.8)	610 (21.1)	

a There were 30,239 participants in the REGARDS study; 56 anomalies were excluded, 2,906 were excluded for missing geocodes, 8.090 for missing dietary information, and 8 for missing covariates, which left 19,179 for the current analysis. RUCA, rural-ruban commuting area; REGARDS, REasons for Geographic and Racial Differences in Stroke

b Southeast: Louisiana, Arkansas, Mississippi, Alabama, Tennessee, Georgia, North Carolina, South Carolina

**Table 3.** The association between residence in a food desert census tract and adherence to the Southern, Plant-based, and Mediterranean dietary patterns in the REasons for Geographic and Racial Differences in Stroke (REGARDS) Study

	Southern Pattern	Plant Based Pattern	Mediterranean Pattern
	OR(95% CI)	OR^a^(95% CI)	OR^a^(95% CI)
**No Stratification**^a^			
All Participants	-	-	0.86 (0.78–0.94)
**Stratified by Race & Education**^a^			
*White*			
Less than High School	1.24 (0.95–1.63)^c^	0.97 (0.72–1.30)	0.81 (0.59–1.12)
High School Graduate	1.41 (1.20–1.67)^c^	0.88 (0.74–1.05)	0.93 (0.78–1.10)
College Graduate and Above	1.91 (1.55–2.35)^c^	0.87 (0.71–1.05)	0.88 (0.73–1.06)
*Black*			
Less than High School	0.90 (0.71–1.15)^c^	1.13 (0.87–1.47)	0.75 (0.56–1.01)
High School Graduate	1.02 (0.89–1.18)^c^	1.03 (0.89–1.21)	0.85 (0.73–1.00)
College Graduate and Above	1.38 (1.14–1.68)^c^	1.01 (0.84–1.22)	0.80 (0.67–0.97)
**Stratified by Region**^b^			
Residence in the southeast	1.25 (1.06–1.46)	1.05 (0.93–1.18)^d^	0.90 (0.80–1.02)
Residence outside the southeast	1.22 (1.08–1.37)	0.85 (0.72–0.99)^d^	0.78 (0.67–0.92)

Abbreviations: OR, odds ratio; CI, confidence interval

Note: Unless indicated, interactions between food desert residence and covariates (race, education, and region) were not statistically significant. If interaction was present, overall associations were not presented.

a The reference group for all ORs is participants in the respective strata, who do not live in food deserts. Models adjusted for age, sex, race, income, education, region, RUCA, relationship status

b Adjusted for age, sex, race, income, education, RUCA, relationship status

c Significant race-food desert interaction, p=0.0005; Significant education-food desert interaction, p=0.003

d Significant region-food desert interaction, p=0.03
